# Structure-emission relationship of some coumarin laser dyes and related molecules: Prediction of radiative energy dissipation and the intersystem crossing rate constants

**DOI:** 10.55730/1300-0527.3526

**Published:** 2022-11-18

**Authors:** M. S. A. ABDEL-MOTTALEB

**Affiliations:** Department of Chemistry, Faculty of Science, Computational Chemistry Lab, Ain Shams University, Cairo, Egypt

**Keywords:** Fluorescence, intersystem crossing, phosphorescence, lifetime, triplet-state loss

## Abstract

Dye lasers are commonly used in optical investigation because their solutions in organic solvents deliver tunable, coherent emissions. They exhibit intense fluorescence owing to some specific spectroscopic characteristics. One drawback of the laser dyes is that it shows excessive triplet-state losses (TSLs.) The lack of theoretical predictions of fluorescence rates, intersystem crossing (ISC), and phosphorescence in laser dyes prompted us to report on the predicted rates of radiative and nonradiative transitions of some laser dyes. Structural engineering by some substituents influencing the simulated rates of coumarin laser dye derivatives for an efficient operation was investigated. The NH_2_ functional group renders the coumarin 120 more fluorescents with reduced TLS than the other investigated materials. Tailoring new efficient laser dyes can be achieved guided by the calculated rates of emission and nonradiative processes.

## 1. Introduction

Coumarin is one of the very famous organic fluorescent materials. Coumarin derivatives have been widely applied in many fields, such as optical brightening agents, photobiological energy transfer processes, light-emitting devices (LEDs) (with the advantages of a sizeable conjugated system and rigid planar structure), laser dyes, medicine, and bio/chemosensors [1–3]. Furthermore, coumarin and its derivatives are well-known laser dyes and valuable in chemical and photochemical studies. Most coumarins are highly fluorescent and have potential applications as fluorescent indicators, sunburn preventives, estimation of enzymes, and high-efficiency dye-sensitized solar cells, among other applications [4–12]. Photophysical characteristics of some coumarin derivatives have been investigated extensively [13–28]. The spectroscopic properties of coumarins have received considerable theoretical and experimental attention due to their ability to lase in the blue-green region [29–31]. Moreover, previous research based on the results of fluorescence quantum yield and lifetime of some coumarin laser dyes of the intramolecular charge transfer type, the interplay between dye structure and solvent properties, which govern the competition between radiative and nonradiative decay, is a subtle function of coumarin structure, solvent properties, and temperature are susceptible to substituent effects. Despite the many theoretical and experimental contributions made on the subject, it is pretty evident that there is still uncertainty about the dominant relaxation mechanism, specifically about the relative contributions of each of the possible internal relaxation channels present in a molecule to the nonradiative decay rate [32].

**Figure 1:**
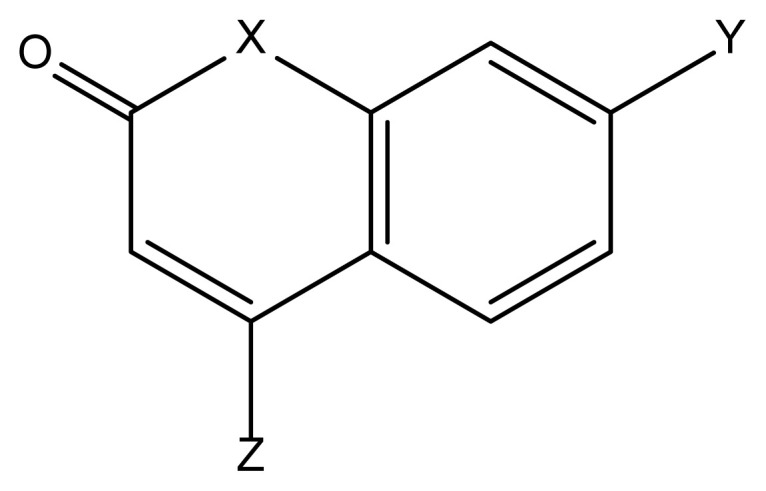
2D structure of the coumarins (1,3,4,5) and related molecule (2) (see Table 1). 3D designs are shown in Figure 2.

Previous work noticed that we still need theoretically predicted spectroscopic characteristics of efficient laser dyes [33]. This paper aims, for the first time, to simulate the missing excited-state dynamic parameters such as fluorescence, ISC, and phosphorescence rates of some coumarin laser dyes induced by different substituents, identified in Figure 1 and Table 1. The varying photophysical properties of coumarin and its various derivatives are sources of motivation that prompted us to study the architecture of the coumarin derivatives, which imparts changes in the electronic distribution within the molecular skeleton. The effect of substituents on the excited state dynamic rates will be investigated by predicting the rates of fluorescence (k_flu_), intersystem crossing (k_ISC_), and phosphorescence (k_Pho_) in the laser dyes and related molecule (**2**) given in Table 1. The current study results will pave the way to highlight the molecular architecture that enables the designing of efficient laser dyes.

## 2. Computational method

All calculations were carried out using the ORCA 4.2 (parallel) software package [34]. Structures were optimized using the B3LYP functional and the def2-TZVP(-F) basis, as recommended [35,36]. The rates and spectra were calculated using the same methods recommended and detailed in the literature [34–36] and summarized here. Ground state calculations were performed for triplet states by setting the multiplicity to three rather than computing the triplet excited states from TD-DFT. To accelerate the computation of two-electron integrals, the resolution of identity approximation was used for the Coulomb part (RIJ) and the chain of spheres algorithm for the exchange part (COSX), with the corresponding auxiliary basis and grid settings [36]. The DFT grid was set to GRID5, and the COSX grid was GRIDX5. ORCA allows vibronic coupling or the so-called Herzberg-Teller (HT) effect by setting the DOHT keyword to true (See the supplementary file). The spin-orbit (SO) coupling integrals were calculated using the RI-SOMF(1X) approximation [36]. For the excited states, TD-DFT no optimized structure presented negative frequencies. The individual rates were calculated using the ORCA_ESD module impeded in the ORCA package. The temperature was set to 77 K. Further details are defined explicitly in the Supplementary material file.

We considered the triplet spin-sublevels (1, 0, or −1). For the molecules under investigation, we predicted the k_ISC_ as the mean of the sum of the individual k_ISC_ (T1), k_ISC_ (T2), and k_ISC_ (T3). A detailed example illustrating the methodology we followed is given in the supplementary file. We employed the well-known conductor-like polarizable continuum (C-PCM) solvation model as treated in ORCA [34] using ethanol (dielectric constant of 24.3 value impeded in ORCA) as a solvent. The supplementary material file gives more details and several examples of calculating the spectra and rates for a molecule.

## 3. Results and discussion

**Figure 2:**
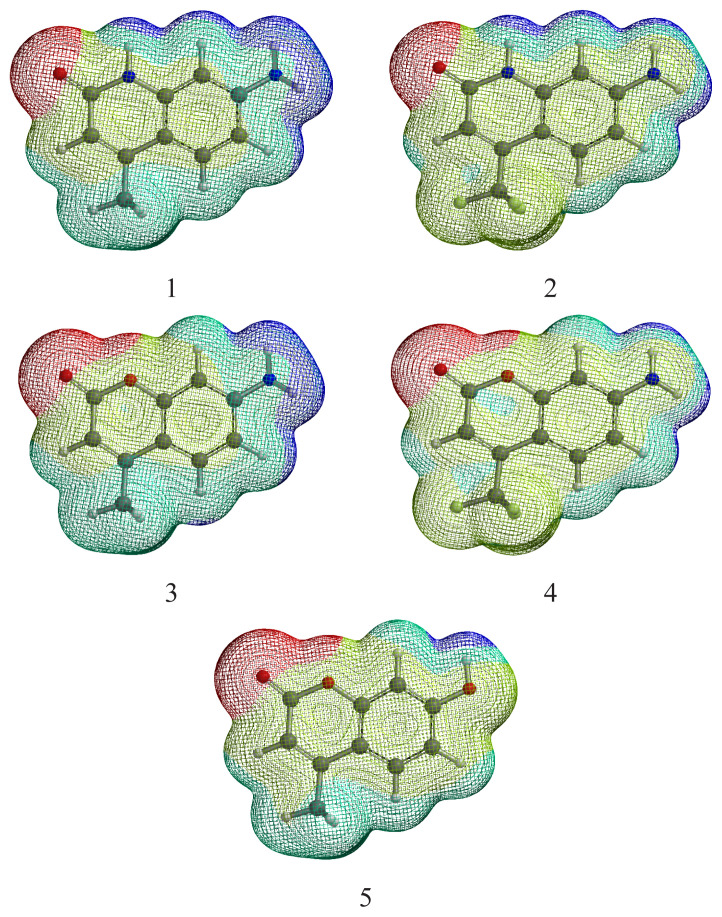
3D structure and PES in the S_0_ state. Color code: blue is the electron-deficient site (positive kJ value), and the red region is the electron-rich region (negative kJ value).

The effect of substituents on the dipole moment is summarized in Table 2. The theoretically simulated photophysical parameters are summarized in Table 3. Figure 2 represents the ground state potential energy surfaces (PES) mapped with the electron density. The electrostatic potential limits in kJ are given in Table 2 for comparison.

**Figure 3:**
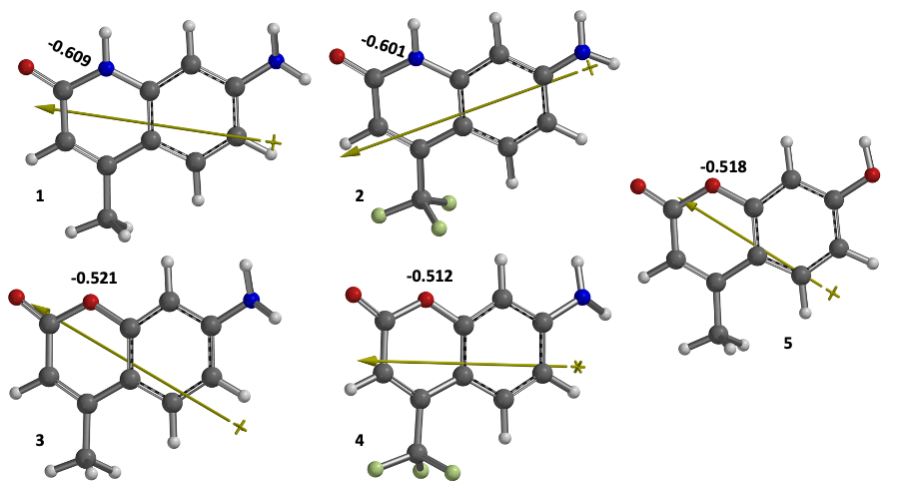
The calculated dipole moment vector directions and the natural charges on the heteroatoms (N and O).

Referring to Figure 1, Table 1, and Table 2, architecturally, three important modified positions are observed in the coumarin skeleton for controlling the photophysical properties. One is the 1- position (X in Figure 1), the second is 7-position (Y in Figure 1), and the third is the 4-position (Z in Figure 1). In this framework, the molecular engineering of these three positions by sensible substituent effect to understand the dominant deactivation channel and the laser efficiency of coumarin derivatives is addressable. Herein, two members of coumarin derivatives with O and N atoms substituted at the X-position were selected. For now, the rates of deactivation channels were successfully tuned by introducing substituents with various electronic properties at the Y-position (**OH** and **NH_2_**, Figure 1) and two-electron donor (**CH_3_**) and electron acceptor (**CF_3_**) functional groups at the Z-position. The effect of substituents can be quantitatively measured by the calculated dipole moment of the molecules given in Table 2 and depicted in Figure 3.

**Figure 4:**
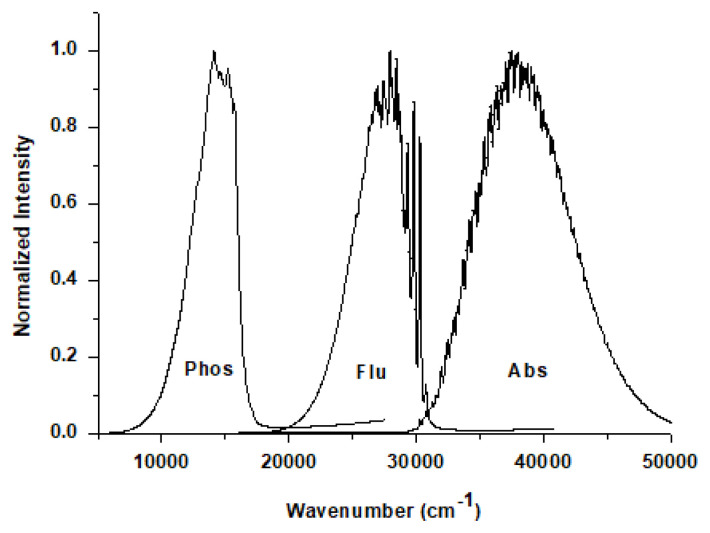
The simulated absorption, fluorescence, and phosphorescence spectra of the laser dye 3 (coumarin 120) in ethanol. Similar spectra were obtained for the other molecules studied.

The predicted spectra of dye 3 (coumarin 120) are depicted in Figure 4 as an example; all other compounds showed similar spectra.

**Figure 5:**
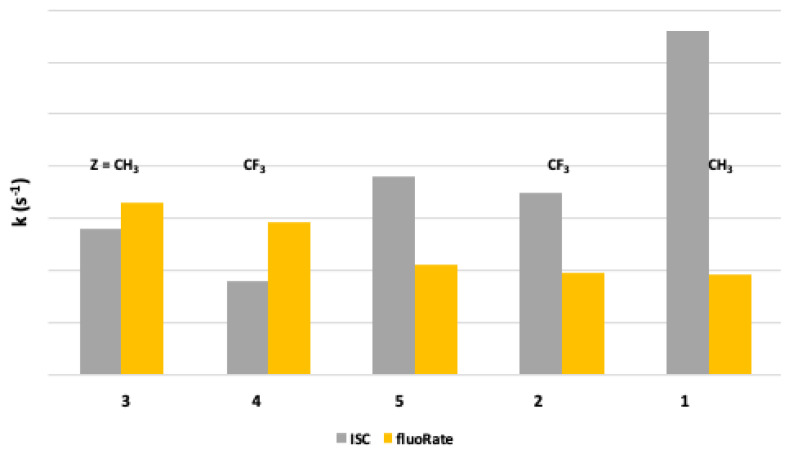
Predicted ISC and fluorescence rates for various molecules at 77 K.

A glance at Figure 5, showing the predicted ISC and fluorescence rates for various molecules at 77 K, reveals the effect of substituents on the nonradiative transition rate k_ISC_ and the radiative k_flu_. Interestingly, a structural change in Y significantly influenced the molecules’ fluorescence, ICS, and phosphorescence rates (see Table 3). One can notice that substituting the OH group in dye 5 (of dipole moment 4.35 Debye) with the more electron-donating NH_2_ group (dye 3, of dipole moment 6.57 Debye) enhances k_flu,_ and k_pho_ slightly depresses k_ICS_. Based on the energies of the fluorescence and the phosphorescence (Table 3), the change in Y by a more powerful electron-donating group could be explained due to lowering the energy gaps between the excited singlet state and the triplet state and the ground state in the case of NH_2_ substituent relative to the OH, resulting in the changes as mentioned above in the rates calculated. Most importantly, the triplet state lifetime is significantly shortened, decreasing light loss by T à T absorption. In other words, the shortening of a triplet-state lifetime due to the replacement of the OH group by the more electron-donating NH_2_ group prevents or limits excessive triplet-state losses (TSLs.) and enhances laser dye efficiency. Moreover, replacing the CH_3_ group in **3** (of 6.57 Debye) with the more electron-withdrawing CF_3_ group in 4 (of 6.33 Debye) further decreases the k_ICS_ value and renders dye 4 less efficient phosphorescent. Consequently, considerable TLS is diminished.

Astonishingly, the N heteroatom in the X position, as in the carbostyryl 7 (dye 1), significantly depresses both k_flu_ and k_pho,_ noticeably enhancing the k_ISC_ value relative to that of dye 3. Consequently, dye 1 should be a less efficient laser dye than dye 3. Molecule 2 behaves similarly to dye 1, indicating that its CF_3_ group is of minor influence, in this case, showing the dominant role the N heteroatom plays in increasing the ISC and decreasing the fluorescence rate. Molecules 1 and 2 are structurally related molecules to the investigated laser dyes. However, the N heteroatom acts as an electron sink (with values of −0.609 and −0.601 natural charges for molecules 1 and 2, respectively) relative to the O heteroatom in coumarins 3, 4, and 5 (having natural charges of −0.521, −0.512, and −0.518, respectively) (See Figure 3).

Noteworthy mentioning is that the experimentally available photophysical data in the literature [4,38–40] match our theoretically predicted parameters (see Table 3). To the best of our knowledge, the triplet state characteristics of all the molecules studied are not reported in the literature [39,40]. Previous experimental research [39,40] rolled out the possibility of ISC based on the absence of empirical proof. However, it was generally assumed that the ISC process is partially responsible for the fast nonradiative deexcitation channel for the fluorescent state of many dyes in nonpolar solvents [39,40].

## 4. Conclusions

For the first time, the research simulates the missing excited-state dynamic parameters such as fluorescence, ISC, and phosphorescence rates of some extensively reported coumarin laser dyes induced by different substituents. Structural substituents that influence the simulated spectroscopic rates of coumarin laser dye derivatives for an efficient operation have been highlighted.

The results showed that all the NH_2_-based dyes (3 and 4) exhibit a much higher fluorescence rate with short-lived triplet-state than those of OH-(dye 5) or -N- heteroatom and NH_2_-based molecules (1 and 2). The results obtained based on the mentioned findings pave the way for designing new efficient laser dyes.

## Supplementary materials

ORCA can compute dynamic properties involving excited states such as absorption spectra, fluorescence, and phosphorescence rates and spectra using the ORCA Excited State Dynamic module. Optimization and frequency calculations should be performed for the ground singlet and triplet states and the excited singlet and triplet states. Hessians are automatically generated, which will be used for generating the spectra and the rates. As an example, full details are given in the supplementary file for the case of dye 4. ORCA manual ( https://orcaforum.kofo.mpg.de/) describes the procedure stepwise. However, we added below some explanations to enable interested researchers to perform their own research using ORCA software.


    # REMARK: AVOGADRO GENERATED ORCA INPUT FILE FOR COUMARIN 4 AS AN EXAMPLE
    ! B3LYP OPT FREQ DEF2-SVP RIJCOSX CPCM(ETHANOL)    **#GS    OPTIMIZATION    AND    HESSIAN   GENERATION**
    %pal nprocs 15 End
    * xyz 0 1
        O              −3.07160             0.27380               −2.06990
        N              4.03680               0.10260               −0.74900
        C              −0.08890              −0.06330            0.66280
        C              0.17310                −0.16110             −0.67210
        C              −1.53380              −0.00940            1.17380
        C              1.07080               0.00960               1.67370
        C              1.63190               −0.07630              −1.16260
        C              −2.52900             0.07610               0.27750
        C              2.64850               0.02310                −0.27240
        C              2.35140               0.04870               1.23660
        C              −2.18570             0.01060               −1.21580
        H              4.12110               −0.39190              −1.61410
        H              4.28760              1.06070                −0.88700
        H              0.86370               0.03490               2.72320
        H              3.15600              0.10040                1.94010
        H              1.84320               −0.10160              −2.21120
        H              −3.54240             0.19680               0.59880
        C              −1.83520             −0.02950              2.68380
        F              −2.96830             0.66330               2.92590
        F              −1.99180              −1.30590            3.09450
        F              −0.80970              0.53660              3.35500
        O              −0.86830             −0.36060             −1.65130
    *
    **# AVOGADRO GENERATED ORCA INPUT FILE TIRPLET GS**
    ! b3lyp opt freq def2-SVP rijcosx AUTOAUX CPCM(ETHANOL)
    %scf ConvForced false END
    MAXITER 300 END
    %GEOM MAXITER 300 END
    %pal nprocs 15 End
    * xyz 0 3
        O            −3.07160           0.27380            −2.06990
        N            4.03680            0.10260            −0.74900
        C            −0.08890            −0.06330         −.66280
        C            0.17310             −0.16110           −0.67210
        C            −1.53380            −0.00940          1.17380
        C            1.07080            0.00960              1.67370
        C            1.63190            −0.07630            −1.16260
        C            −2.52900            0.07610            0.27750
        C            2.64850            0.02310               −0.27240
        C            2.35140            0.04870              1.23660
        C            −2.18570            0.01060             −1.21580
        H            4.12110            −0.39190             −1.61410
        H            4.28760            1.06070               −0.88700
        H            0.86370            0.03490              2.72320
        H            3.15600            0.10040              1.94010
        H            1.84320            −0.10160            −2.21120
        H            −3.54240            0.19680            0.59880
        C            −1.83520            −0.02950          2.68380
        F             −2.96830            0.66330            2.92590
        F             −1.99180            −1.30590          3.09450
        F             −0.80970            0.53660            3.35500
        O             −0.86830            −0.36060           −1.65130
    *
    # AVOGADRO GENERATED ORCA INPUT FILE COUMARIN 4 **EXCITED STATE OPTIMIZATION AND HESSIAN GENERATION**
    ! b3lyp opt def2-SVP rijcosx AUTOAUX CPCM(ETHANOL)
    %TDDFT NROOTS 5
               IROOT 1
    END
    %SCF MAXITER 300 END
    %GEOM MAXITER 300 END
    %pal nprocs 20 end
    * XYZFILE 0 1 Name-GS.xyz **# read the coordinates from the external file Name-GS.xyz**
    # ABSORPTION SPECTRUM GENERATION OF COUMARIN 4 (NAME-ABS) ROOM TEMP
    !ESD(ABS) B3LYP DEF2-SVP RIJCOSX AUTOAUX SLOWCONV TIGHTSCF CPCM(ETHANOL)
    %TDDFT NROOTS 5
               IROOT 1
    END
    %MaxCore 1024
    %scf ConvForced false END
    %SCF MAXITER 300 END
    %GEOM MAXITER 300 END
    %ESD                            GSHESSIAN   “NAME-GS.hess” #HESSIAN FILE GENERATED FROM THE GS COMPUTATIONS
                          ESHESSIAN “NAME-ES.hess” #HESSIAN FILE GENERATED FROM THE ES COMPUTATIONS
                          TCUTFREQ        300
                          IFREQFLAG REMOVE
                          DOHT true           **#** includes vibronic coupling or the so-called Herzberg-Teller (HT) effect
    END
    %pal nprocs 20 End
    * XYZFILE 0 1 Name-GS.xyz

    # PHOSPHORESCENCE SPECTRA AND RATES OF COUMARIN 4 (NAME-PHO0 77K TEMP
    !B3LYP DEF2-TZVP(-F) ESD(PHOSP) TIGHTSCF GRID5 GRIDX5 RIJCOSX RI-SOMF(1X) CPCM(ETHANOL)
    %TDDFT NROOTS 5
                          DOSOC TRUE
                          TDA FALSE
               IROOT 1
    END
    %MaxCore 1024
    %scf ConvForced false END
    %SCF MAXITER 300 END
    %GEOM MAXITER 300 END
    %ESD                         GSHESSIAN “NAME-GS.hess”
                          TSHESSIAN “NAME-TS.hess”
                          TCUTFREQ        100
                          IFREQFLAG REMOVE
                          DOHT true
                          DELE 15800.7 **#The difference between the T1 and GS ENERGY**
                          TEMP 77
    END
    %pal nprocs 20 End
    * XYZFILE 0 1 Name-GS.xyz
    $NEW_JOB
    !B3LYP DEF2-TZVP(-F) ESD(PHOSP) TIGHTSCF GRID5 GRIDX5 RIJCOSX RI-SOMF(1X) CPCM(ETHANOL)
    %TDDFT NROOTS 5
                          DOSOC TRUE
                          TDA FALSE
               IROOT 2
    END
    %MaxCore 1024
    %scf ConvForced false END
    %SCF MAXITER 300 END
    %GEOM MAXITER 300 END
    %ESD                         GSHESSIAN “NAME-GS.hess”
                          TSHESSIAN “NAME-TS.hess”
                          TCUTFREQ        100
                          #IFREQFLAG REMOVE
                          DOHT true
                          DELE 15800.7 #T1 ENERGY
                          TEMP 77
    END
    %pal nprocs 19 End
    * XYZFILE 0 1 Name-GS.xyz
    $NEW_JOB
    !B3LYP DEF2-TZVP(-F) ESD(PHOSP) TIGHTSCF GRID5 GRIDX5 RIJCOSX RI-SOMF(1X) CPCM(ETHANOL)
    %TDDFT NROOTS 5
                          DOSOC TRUE
                          TDA FALSE
               IROOT 3
    END
    %MaxCore 1024
    %scf ConvForced false END
    %SCF MAXITER 300 END
    %GEOM MAXITER 300 END
    %ESD                         GSHESSIAN “NAME-GS.hess”
                          TSHESSIAN “NAME-TS.hess”
                          TCUTFREQ        100
                          IFREQFLAG REMOVE
                          DOHT true
                          DELE 15800.7 #T1 ENERGY
                          TEMP 77
    END
    %pal nprocs 20 end
    * XYZFILE 0 1 Name-GS.xyz
    **# DYE 1-ISC ROOM TEMP**
    !B3LYP DEF2-TZVP(-F) ESD(ISC) TIGHTSCF GRID5 GRIDX5 RIJCOSX RI-SOMF(1X) CPCM(ETHANOL)
    %TDDFT NROOTS 5
               SROOT 1
               TROOT 1
               TROOTSSL 1
               DOSOC TRUE
    END
    %MaxCore 1024
    %scf ConvForced false END
    %SCF MAXITER 300 END
    %GEOM MAXITER 300 END
    %ESD            ISCISHESS “Name-ES.hess”
    ISCFSHESS “Name-TS.hess”
              USEJ TRUE
              DOHT true
              #TEMP 77                           REMOVE # IN CASE OF 77 K CALCULATIONS
              DELE 28643.09
    END
    %pal nprocs 35 END
    * XYZFILE 0 1 Name-TS.xyz
    $NEW_JOB
    !B3LYP DEF2-TZVP(-F) ESD(isc) TIGHTSCF GRID5 GRIDX5 RIJCOSX RI-SOMF(1X) CPCM(ETHANOL)
    %TDDFT NROOTS 5
               SROOT 1
               TROOT 1
               TROOTSSL 0
               DOSOC TRUE
    END
    %MaxCore 1024
    %scf ConvForced false END
    %SCF MAXITER 300 END
    %GEOM MAXITER 300 END
    %ESD ISCISHESS “Name-ES.hess”
              ISCFSHESS “Name-TS.hess”
              USEJ TRUE
              DOHT true
              #TEMP 77
              DELE 28643.09
    END
    %pal nprocs 35 ENS
    * XYZFILE 0 1 Name-TS.xyz


References1S
ORCA 4, 2 manual
at https://orcaforum.kofo.mpg.de/
2S

NeeseF

Software update: the ORCA program system, version 4.0 WIREs
Comput Mol Sci
2018
8
e1327

https://doi:10.1002/wcms.1327

3S

NeeseF

Software update: The ORCA program system— Version 5.0
WIREs Computational Molecular Science
2022
12
e1606
10.1002/wcms.1606


## Figures and Tables

**Table 1:** Dyes studied, ChemSpider ID*, (see Figure 1).

Dye (ChemSpider ID)	X	Y	Z
**1** carbostyryl 7 (79637)	NH	NH_2_	CH_3_
**2** 2-quinolinone (168395)	NH	NH_2_	CF_3_
**3** coumarin 120 (83285)	O	NH_2_	CH_3_
**4** coumarin 151 (90930)	O	NH_2_	CF_3_
**5** coumarin 4 (4444190)	O	OH	CH_3_

* https://www.chemspider.com/StructureSearch.aspx

**Table 2:** Structure-properties correlation of the coumarins which are listed in Table 1.

LASER DYE	Dipole (D)	Max(PES) kJ	Min(PES) kJ
**1**	**6.02**	**263.34**	−**288.4**
**2**	**6.63**	**288.58**	−**254.09**
**3**	**6.57**	**275.12**	−**279.76**
**4**	**6.33**	**295.25**	−**242.58**
**5**	**4.35**	**361.72**	−**259.81**

**Table 3:** Some predicted radiative (S_1_ à S_0_ and T_1_ à S_0_) and nonradiative (ISC) transitions of the dyes 1–5. Phosphorescence lifetime (τ) in ms is given between parentheses. The percent of the Hertzberg-Teller coupling (HT%) due to vibronic coupling is also provided. Wavelength is shown in the linear wavenumber units.

Dye	k_phos_, s^−1^ (, ms)	HT%	λ_phos_, cm^−1^	k_ISC_, 10^9^ s^−1^	k_flu_, 10^9^ s^−1^	λ_flu_, cm^−1^	*k_flu_ 10^9^ s^−1^	*k_nr_ 10^9^ s^−1^
1	0.30 (3333.3)	91.1	14,807	0.33	0.10	25,126	--	--
2	0.62 (1612.9)	98.5	14,598	0.18	0.10	23,529	--	--
4	5.40 (185.2)	99.5	11,854	0.09	0.15	22,883	3.65 [37]	54.3 [38]
5	5.80 (172.4)	99.5	16,525	0.19	0.11	26,810	0.22 [38]	0.94 [39]
3	294 (3.40)	99.9	14,116	0.14	0.17	24,331	0.12 [39]	1.24 [40]
